# OTULIN-related conditions: Report of a new case and review of the literature using GenIA

**DOI:** 10.21203/rs.3.rs-3950863/v2

**Published:** 2024-03-08

**Authors:** Andrés Caballero-Oteyza, Laura Crisponi, Xiao P. Peng, Hongying Wang, Pavla Mrovecova, Stefania Olla, Chiara Siguri, Farida Marnissi, Zineb Jouhadi, Ivona Aksentijevich, Bodo Grimbacher, Michele Proietti

**Affiliations:** 1. Clinic for Immunology and Rheumatology, Hanover Medical School, Hanover, Germany; 2. Institute for Immunodeficiency, Center for Chronic Immunodeficiency, University Hospital Freiburg, Freiburg, Germany; 3. RESiST-Cluster of Excellence 2155, Hanover Medical School, Hanover, Germany; 4. Institute for Genetic and Biomedical Research (IRGB), The National Research Council (CNR), Monserrato, Cagliari, Italy; 5. Department of Genetic Medicine, Johns Hopkins School of Medicine, Baltimore, Maryland, USA; 6. Inflammatory Disease Section, National Human Genome Research Institute, Bethesda, Maryland, USA.; 7. Pathology Center university hospital, Ibn Rochd, University Hassan 2, Casablanca, Morocco.; 8. Laboratory of Cellular and Molecular Pathology, Immunopathology of Infectious and System Diseases, Department of Infectious Diseases, University Children’s Hospital Ibn Rochd, University Hassan 2, Casablanca, Morocco; 9. Clinic of Rheumatology and Clinical Immunology, Center for Chronic Immunodeficiency (CCI), Medical Center, Faculty of Medicine, Albert-Ludwigs-University of Freiburg, Germany; 10. RESiST – Cluster of Excellence 2155 to Hanover Medical School, Satellite Center Freiburg, Germany; 11. DZIF – German Center for Infection Research, Satellite Center Freiburg, Germany; 12. CIBSS – Centre for Integrative Biological Signaling Studies, Albert-Ludwigs University, Freiburg, Germany

**Keywords:** Systematic review, OTULIN, ORAS, IMD107, OTULIN haploinsufficiency, autoinflammation, immunodeficiency, ubiquitin, NF-**κ**B, GenIA, human genetics

## Abstract

*OTULIN* encodes an eponymous linear deubiquitinase (DUB), which through the regulation of M1-Ub dynamics, is essential for controlling inflammation as a negative regulator of the canonical NF-**κ**B signaling pathway. Biallelic loss-of-function (LOF) mutations in *OTULIN* cause an autosomal recessive condition named Otulin-Related Autoinflammatory Syndrome (ORAS), also known as Otulipenia or AutoInflammation, Panniculitis, and Dermatosis Syndrome (AIPDS). Monoallelic *OTULIN* LOF, also known as OTULIN Haploinsufficiency (OHI) or Immunodeficiency 107 (IMD107), has been linked to an incompletely penetrant, dominantly inherited susceptibility to invasive Staphylococcal infections. At the same time, a recent novel ORAS-like inflammatory syndrome was described in association with a heterozygous missense mutation that appears to exert dominant negative effects. In this manuscript, we report the identification of a novel homozygous missense mutation, c.595T>A; p.(Trp199Arg), in a Moroccan infant with an ORAS phenotype. We go on to systematically review the literature for OTULIN-related human disease phenotypes by using the GenIA database to collect, extract and harmonize all clinical, laboratory and functional data for published patients and variants. Our comprehensive synthesis of genotypic, phenotypic, and mechanistic data enables a more in-depth view of the diverse mechanisms and pathways by which the *OTULIN* pathogenic variants may lead to human immune disease. This review may help variant classification activities and the drafting of diagnostic and management guidelines; but it also identifies outstanding knowledge gaps and raises additional questions for future investigation.

## Introduction

Monogenic systemic autoinflammatory disorders (SAIDs) encompass one of the fastest growing categories of genetically-driven immune disease. In particular, mutations in genes that regulate ubiquitin (Ub) signaling have been associated with diverse Mendelian diseases, many featuring immune and inflammatory phenotypes. Ubiquitination is a dynamic and complex post-translational modification (PTM) that influences all cellular processes by regulating protein turnover, activity, and subcellular localization [1]. Ub monomers or chains are attached to protein targets through the combined action of E1, E2, and E3 ligases and are removed by deubiquitinases (DUBs).

The cellular ubiquitin pool is in a constant state of flux, with many potential combinations of polymeric linkages and substrates leading to diverse signaling and cellular outcomes. Met1-linked linear polyubiquitination (M1-Ub) is particularly important for regulating cell-intrinsic immune responses, such as those involving NF-**κ**B signaling. Upstream activation of TNF or IL-1 receptors triggers the formation of a signaling complex that involves linear ubiquitination of various target proteins to facilitate activation of the canonical NF-**κ**B pathway, which then drives nuclear transactivation of genes involved in inflammation, cell proliferation, and cell survival [2–5].

Dynamic regulation of M1-Ub chain assembly and disassembly involves balancing the action of the linear ubiquitin chain assembly complex (LUBAC), which comprises HOIP, HOIL-1 and SHARPIN [6,7], with that of DUBs such as OTULIN. LUBAC is the only Ub E3 ligase known to generate M1-Ub chains and as such, it is required for full activation of the inhibitor of **κ**B (IκB) kinase (IKK) complex, leading to I**κ**B phosphorylation and degradation with consequent NF-**κ**B/p65 derepression, activation, and nuclear translocation. LUBAC is also recruited to the TNF receptor (TNFR) signaling complex to modify RIPK1 and NEMO with M1-Ub chains, leading to NF-**κ**B activation and inhibition of cell death [8].

Both OTULIN and CYLD can disassemble M1-Ub chains, but OTULIN is the only known vertebrate DUB with exclusive specificity for M1-Ub chains [9,10], while full-length CYLD preferentially cleaves K63-linked chains [11]. Both DUBs interact with the LUBAC complex - OTULIN directly binds the PUB domain of the catalytic subunit HOIP, which then recruits it to the TNFR complex. OTULIN DUB activity reverses LUBAC-dependent effects via M1-Ub chain removal from targets such as NEMO, RIPK1, ASC, and TNFR1, but it also activates LUBAC by removing auto-inhibitory M1-Ub chains from LUBAC components [12]. OTULIN suppresses NF-**κ**B signaling by binding LUBAC and removing I**κ**B-bound M1-Ub chains via the catalytic OTU domain (OTU-cat).

*Otulin* knockout (KO) mice show embryonic lethality with evidence of acute systemic inflammation and excessive cellular accumulation of linear polyubiquitin chains, while inducible LOF in adult mice also leads to a pro-inflammatory cytokinopathy with increased cell death and tissue degeneration in bone marrow, thymus, liver, small intestine, and heart [13]. Mice with OTULIN deficiency in myeloid cells develop spontaneous inflammation, while those with OTULIN deficiency in B or T cells are healthy. Conditional OTULIN loss in keratinocytes leads to the development of inflammatory skin lesions driven by TNFR1-mediated, RIPK1-dependent keratinocyte death, primarily via necroptosis [14].

Mutations in all three subunits of LUBAC, along with NEMO and RIPK1, may be associated with predominantly autoinflammatory disease in animal models and humans [15–18]. OTULIN has recently been associated with 3 immune conditions that present with distinct clinical features and inheritance patterns. Biallelic OTULIN LOF mutations are associated with ORAS [OMIM#6170990], while heterozygotes with OHI have increased susceptibility to invasive *Staphylococcus aureus* infection [OMIM#619986]. More recent reports suggest that severe ORAS-like disease can also arise from dominant-negative mutations. Mechanistically, the inability of OTULIN to remove M1-Ub chains from key substrates mentioned above results in disrupted Ub pool dynamics and/or constitutive activation of inflammatory signaling pathways such as NF-**κ**B and type I interferon (IFN) [19].

Herein, we describe the case of a Moroccan infant with severe ORAS associated with a novel homozygous *OTULIN* missense mutation: p.(Trp199Arg), and present a detailed review of the current literature on OTULIN-related diseases using the recently developed GenIA (Genetic Immunology Advisor) database [20].

## Materials and Methods

### Diagnostic Genetic Testing

Whole-exome sequencing (WES) and subsequent analysis was performed from peripheral blood DNA as previously described [21]. Sanger sequencing was used for confirming segregation in family members. In this manuscript, we use the term ‘mutation’ for sake of expediency but recognize that the field of human genetics is endeavoring to move away from this term towards the use of more appropriate terms such as ‘Pathogenic/Likely Pathogenic variant’.

### *In silico* 3D modeling

We used the X-ray crystallography structure (PDB ID: 4KSJ) of the wild-type (WT) OTU domain obtained at 1.6 Å resolution [10]. Maestro software (Schrödinger Release 2022–3) was used to generate the mutated form of the protein by replacing tryptophan-199 with arginine (W199R). Both WT and mutant proteins were then prepared using the Protein Preparation Wizard tool [22]. We used the Adaptive Poisson–Boltzmann Solver (APBS) [23] integrated into PyMOL (Version 2.5.5, Schrödinger, LLC.) to compare the solvation and electrostatics of WT vs mutant protein. Prior to map generation, structural preparations were conducted using the pdb2pqr method [24], and the outcomes were visually represented through a color-coded surface. Molecular dynamics simulations (MD) were executed using Desmond [25]. For both simulations, we used the TIP3P solvent model [26] and an OPLS4 force field, a 10.0 Å orthorhombic water box, subjected to minimization and neutralization by the addition of ions (Na+ or Cl-). Both simulations lasted 500 ns, and trajectories were recorded every 100 ps, within the NPT ensemble. Temperature (300.0 K) and pressure (1.01325 bar) were maintained constant using the Nosé-Hoover thermostat [27] and the Martyna-Tobias-Klein barostat [28] methods, respectively. Other parameters remained at their default settings. The results were analyzed using the Simulation Interaction Diagram tool integrated into Desmond [25]. Finally, DUET [29] was used to examine *in silico* effects of the W199R mutation on protein stability.

### Cell transfections, luciferase and deubiquitinase assays

LUBAC plasmids (mixed equal amounts of 3xFLAG-HOIP, 3xFLAG-tag-HOIL-1, 3xFLAG-tag-SHARPIN, obtained from Addgene #50014, #50015, #50016) [30], OTULIN WT with MYC-FLAG tag (Origene# RC224840) or mutant plasmid generated by site-directed mutagenesis kit (Agilent), empty vector, together with equal amount NF-kB driven luciferase reporter plasmid/renilla control plasmid (Promega) were transfected into HEK293T cells in 24-well plates. After 18 hr in culture, 1/4th of HEK293T cells were collected and subjected to dual-luciferase assay according to Promega’s protocol. The fold change of Firefly luciferase versus Renilla luciferase for each of the transfectants was then normalized to cells transfected with an empty vector. Cell lysates were collected and subjected to Western blotting using antibodies against OTULIN, SHARPIN, HOIL-1, HOIP, and loading control GAPDH (SCBT: Myc-tag, SC-40; Cell Signaling: Sharpin, #12541; FLAG-tag, #14793; GAPDH, #2118). For the deubiquitinase assay [33], LUBAC plasmids, OTULIN WT or mutant OTULIN plasmid, together with pEF-Nemo[31] and HA-Ub (Addgene#17608), were transfected into HEK293T cells in 6-well plates and cultured for 48hrs. Cells lysates were immunoprecipitated with anti-NEMO antibody (SCBT sc-8032), then blotted with anti-NEMO (Cell Signaling #2685), anti-Myc (Cell Signaling #2278) and anti-HA (Cell Signaling #3724) antibodies. RNA was extracted using Purelink RNA mini kit with on-column DNase digestion (Thermo Fisher). After Turbo-DNase I treatment, 2 μg RNA were retro-transcribed into cDNA with the SuperscriptIV VILO (Thermo Fisher). Primers for *OTULIN* and *GAPDH* were designed specifically to bind exon-exon junctions using Primer3 (sequences available upon request). 5% of the generated cDNA was used for amplification with the GeneAmp Fast PCR kit (Thermo Fisher). PCR products were loaded onto an agarose gel.

### Systematic review using GenIA

To systematically review current knowledge and available information about OTULIN-related diseases, we used the GenIA database [20]. GenIA uses a patient-centered model to connect structured, harmonized datasets containing genotypic, phenotypic and mechanistic information. Through the data entry forms in the GenIA curator portal, we systematically registered all available genetic, clinical, immunophenotypic, therapeutic and functional data for all the patients reported so far in the literature, as well as the novel one, and their family members. We additionally collected all available experimental or *in silico* functional data generated for OTULIN variants (Figure S1). Once finished with data collection, we mined GenIA’s website using the *Gene Search* module to obtain the list of *OTULIN* associated genetic conditions with their respective modes of inheritance, mechanisms of action, and number of reported patients and families. For each condition, we then extracted all known patients and family members (Table S1) along with their respective pedigrees. For each individual, we obtained demographics, clinical findings (based on HPO terms), clinical laboratory studies, therapies tried, and patient cell-derived assay data. For each variant, we extracted the following data: gene and chromosome location; (predicted or confirmed) cDNA and protein change; frequencies in healthy population databases such as gnomAD; links to external resources (i.e. dbSNP, ClinVar, OMIM or UniProt); clinical and functional classifications based on ACMG criteria; individuals and families carrying each variant with associated zygosity information; and results of *in vitro* functional characterization. We compiled and harmonized the above data into a review of the current literature about OTULIN-related diseases, focusing on novel mechanistic observations that may shed light on genotype-phenotype relationships.

### Data analysis and visualization

We used the R programming language through RStudio for data analysis and visualization, as well as table and graph generation. Affinity Designer software was used to create figures.

## Results

### Case presentation and functional studies

Our index patient (M107) was born to consanguineous healthy parents of Moroccan ancestry (Figure 1A) and hospitalized soon after birth for failure to thrive with clinical and laboratory evidence of severe, sterile systemic inflammation (Figure 1B, Suppl. Material). Her disease progressed despite high-dose steroids and broad-spectrum antimicrobials (Suppl. Material). A recessive disorder was suspected and WES identified a homozygous *OTULIN* variant [ENST00000284274.5: c.595T>A; p.(Trp199Arg), henceforth referred to as W199R] consistent with the patient’s presentation and segregating appropriately in her parents (Figure 1A,C). This variant is absent from large population databases such as gnomAD v.4.0, has not been previously reported in the literature or in large variant databases such as ClinVar, is predicted to result in the non-conservative substitution of a highly conserved OTU domain residue, and is considered deleterious by multiple *in silico* algorithms.

Unfortunately, our index patient passed away before any functional studies could be performed. Therefore, we performed *in silico* comparative 3D structural modeling of WT and W199R OTULIN and observed that the latter alters a number of intra-protein interactions (Figure 1D, S2). Specifically, hydrogen bonds involving Leu195, Ala203, and Leu202 are lost, while those with Gly144 and His300 are gained, while hydrophobic and/or aromatic interactions involving Ala138, Met139, Ala142, Pro146, Trp148 and Leu149 are reduced. W199R also results in greater protein instability with a ΔΔG (change in Gibbs free energy) of - 2.061 Kcal/mol [29] and a change in electrostatic potential via increased positive surface area (Figure S2A), which may affect interactions with other proteins. Additionally, MD modeling studies found increased dynamic fluctuation (RMSF) of catalytic core-proximal loop residues 281–285, which are involved in polyUb interaction (Figure S2B-C), so OTULIN’s affinity for polyUb may also be affected.

To directly address the functional consequences of W199R, we cloned and transfected this variant into HEK293 cells and observed severely reduced (~75%) protein levels of OTULIN-W199R relative to WT or the known pathogenic OTULIN-L272P variant (Figure 1E,G, S2D). This may be attributable to a splicing defect leading to mRNA instability (c.595T is the first nucleotide of exon 6) and/or to the protein instability mentioned above. Since mRNA levels of this variant expressed in HEK293T cells remained stable (Figure S2E), we concluded that the latter is more likely. The increased Ub chain accumulation seen after NEMO immunoprecipitation shows that both OTULIN-W199R and -L272P mutants failed to deubiquitinate NEMO to the same extent as the WT protein *in vitro* (Figure 1F). The higher mean ubiquitination intensity observed when overexpressing NEMO + HA-Ub in the absence of LUBAC (Figure 1F, S2F) is likely attributable to retention of all Lysine residues on the Ub, although this signal may also come from other types of ubiquitination. As additional support for its pathogenicity, OTULIN-W199R led to increased NF-kB activity by luciferase reporter assay to the same extent as the known LOF variant OTULIN-L272P (Figure 1H).

### Systematic literature review and genetics

Our comprehensive literature search identified 13 relevant research/review articles (12 from PubMed and one from MedRxiv) reporting three OTULIN-related conditions [9,13,19,32–41]. In total (including our Moroccan family), we identified 16 families and 116 individuals (Table S1, Figure S3), 56 of whom carry monoallelic or biallelic variants (Table 1). Among these 56 individuals, 9 from 7 families were reported to have ORAS - with 8 homozygotes and 1 compound heterozygote. An additional 16 individuals from 10 families were diagnosed with OHI, including subjects H077 and H079, who were obligate/presumed heterozygotes for the familial variant, but also including the recently reported patient with a heterozygous p.(A240V) variant, who appears to have had a mechanically triggered, sterile inflammation [41]. Finally, we assigned the diagnosis of DN-ORAS to 2 individuals from 2 families, who shared the same dominant-negative catalytic domain variant p.(C129S). The remaining 29 individuals harbored variants but were unaffected (Table 1).

As previously noted [20], the same patients and family members may be reported in more than one independent study, so we examined the existing literature for such redundancies and found that 1 patient had been included in 4 articles (A023), 6 patients in 3, and 31 individuals in 2 (Figure 2A). Of the 28 reported patients, 27 were genetically confirmed, 26 had detailed clinical data available, 16 had functional data performed on their primary cells, and 10 had available immunophenotyping data. However, all four datasets were present for only 7 patients (Figure 2B).

For this study, we evaluated all 38 reported variants (including our patient’s novel W199R and the 2 variants reported as Likely Pathogenic in ClinVar) (Table 2). Unfortunately, we could not confirm the appropriate cDNA information for the variant “TQK100–102AAA” reported by Keusekotten et al. [9] or for the variant “c.395_396ins, p.Leu131_Arg132insLeuCysThrGlu” reported by Gezgin et al. [38], so these cannot be included until we receive further details from the authors. Of note, 10 of the 16 disease-associated variants reported to date could not be found in OMIM or ClinVar (Figure 2A).

The OTULIN protein consists of an N-terminal PUB-interacting motif (PIM) domain, an ovarian tumor (OTU) domain and a C-terminal PDZ binding motif (Figure 2C). All 16 mutations associated with OTULIN-related diseases (Figure 2C), including W199R, are located within the large OTU domain (aa79–348), which is required for M1-Ub chain binding and hydrolysis. In particular, conformational regulation of the catalytic triad Cys129-His339-Asp341 within this domain is important for determining OTULIN function and specificity [42]. Twelve missense mutations are associated with either DN-ORAS or ORAS and/or OHI; 2 frameshift mutations are associated with ORAS and/or OHI; 1 stop codon is associated with OHI; and 1 splice-altering variant [c.864+2T>C; (EX6+2T>C); p.(W199_Q288del)] associated with both ORAS and OHI results in the production of smaller transcripts corresponding to skipping of exons 5 and/or 6 or retention of 17 nucleotides between exons 4 and 5 followed by exon 6 skipping [34]. Thus, these mutations may affect OTULIN catalytic activity, its binding to linear chains and/or protein stability.

### Clinical features and treatment outcomes of OTULIN-related diseases

We compared clinical phenotypes across the 9 ORAS, 16 OHI and 2 DN-ORAS patients (Figure 3A). Although the number of reported patients for each condition was limited, we can see that all ORAS or DN-ORAS patients, regardless of mutational mechanism, were affected by various manifestations of systemic autoinflammation. These included but were not limited to recurrent fevers, arthritis/arthralgias, diarrhea, lipodystrophy and erythematous rashes with painful subcutaneous nodules (panniculitis) (Figure 3A). Laboratory studies were notable for elevated inflammatory markers, leukocytosis and neutrophilia in the absence of known infection, as well as evidence of neutrophilic skin infiltration on histopathology. No evidence of immunodeficiency in these patients was reported/described. Of note, compound heterozygosity for 2 hypomorphic mutations has been associated with later-onset disease characterized by life-threatening, multi-organ sterile abscesses involving the skin, lung, and spleen [37]. When all ORAS and DN-ORAS patients are considered (12 total), we noted a mean diagnostic delay of ~7 years between symptom onset and achievement of molecular diagnosis (Figure 3B).

Significantly more phenotypic variability is seen for OHI. Most patients with OHI experience their first infection episode during adolescence [39], with some more severely affected than others. Clinical involvement for many parents of ORAS patients may be subtle and may not be revealed without dedicated clinical re-evaluation [13,32,34,35]. Levels and functions of immune cells when measured appear normal, supporting the hypothesis that the molecularly relevant defect may reside in non-hematopoietic cells [39]. Transmission of OHI in the families reported was consistent with autosomal dominant inheritance with variable expressivity and incomplete penetrance. We calculated that 38.2% of confirmed heterozygotes are clinically affected, but this penetrance estimate decreases to 34.9% with the inclusion of presumed/obligate heterozygous carriers for pathogenic variants (Figure 3C).

Regarding management outcomes, data were available for 11 individuals from 10 families, including 2 DN-ORAS cases (Figure 3D, Table S2). All reported patients received steroids and all except our ORAS patient (M107) also received at least one form of immunomodulation (up to a maximum of 3 different classes). The most common agent used was TNF inhibitor (n = 7), followed by IL-1 inhibitor/anakinra (n = 5) and methotrexate (n = 4), azathioprine (n = 2), and colchicine or JAK inhibitor/ruxolitinib (n = 1). Almost all individuals responded positively to steroids to some extent but many went on to be trialed with other agents. All 7 patients who received TNF inhibition (four ORAS and three DN-ORAS) showed at least moderate, if not robustly, positive responses. By comparison, anakinra, colchicine, ruxolitinib and methotrexate elicited only partial clinical responses in one individual at most. One patient ultimately received a curative hematopoietic stem cell transplant (HSCT) after failing several kinds of immunomodulation (Figure 3D, Table S2). By contrast, the use of antimicrobials in the non-OHI patients was much less frequently reported (n = 4), with known or positive outcomes in only one patient treated for pneumonia (not shown).

### Molecular and cellular consequences of OTULIN LOF

#### Patient cell data

For 7 ORAS patients, 8 OHI patients, and 2 unaffected heterozygotes (F066, F067), more detailed cellular characterization at baseline or in response to specific stimuli was available (Figure S4, Table S3). Levels of *OTULIN* mRNA were comparable to controls for 8/11 tested patients and decreased for 1 ORAS (C039) and 2 OHI (H080 and H083) patients [Row 1]. Expectedly, cDNA sequence was abnormal for 2 individuals (E062 and E065) sharing a splice-altering variant [Row 2]. All patients except one with DN-ORAS (N110) showed decreased or nearly absent OTULIN protein levels [Row 3]. Protein (and mRNA) levels of LUBAC components (HOIP, HOIL-1 and SHARPIN) were comparable to controls in some tested patients but mildly to clearly reduced in others, with low expression of one, two or all three components [Rows 4–9].

Fibroblasts or immune cells from all tested individuals showed increased accumulation of M1-Ub chains, consistent with loss of OTULIN-mediated deubiquitination [Rows 11–12] [9]. Fibroblasts and PBMCs from 3 ORAS patients (A023, B035, C039) also showed increased linear ubiquitination of specific targets such as NEMO, TNFR1, RIPK1 and ASC under stimulation by TNF or IL-1β [32]. Moreover, fibroblasts from 6 ORAS or OHI patients showed accumulation of high molecular weight caveolin-1 complexes, presumed or shown to be modified by K63-Ub [Rows 13–14].

Stimulated fibroblasts or T cells from ORAS patients (A023, B035, C039) showed increased phosphorylation of JNK, IKKα/β, I**κ**B-α, p38, p65-NF-**κ**B, suggesting activated NF-**κ**B and MAPK signaling [Rows 16–21], along with increased expression of pro-inflammatory cytokines (i.e. IL-18, TNF-α, IL-6, IL-12, IL-1β), and increased production of IFN-γ [Rows 22–34] (Figure 4).

PBMCs and fibroblasts from 3 ORAS patients showed reduced caspase-like proteasome activity relative to controls, as well as downstream abnormalities in proteasome assembly and function [Rows 35–39], leading to accumulation of unfolded or K48-ubiquitinated proteins [Row 40]. Tryptic-like and chymotryptic-like proteasome activity was reduced in PBMCs but not fibroblasts from 2/3 patients, specifically implicating the immunoproteasome, a known OTULIN substrate [19]. This was associated with increased expression of IFN-stimulated genes in DN-ORAS (N110) and ORAS (C039) patients [Rows 41–54], along with increased levels of IFN-α and other inflammatory signals (i.e. IP10, MIG, RANTES, MCP1) in the PBMCs, monocytes, whole blood or serum of ORAS patients (A023, B035, C039) [Rows 55–60].

Finally, ORAS and DN-ORAS skin fibroblasts or hepatocytes (A027) showed increased apoptosis after stimulation by TNF+BV6, TNF+CHX, alpha-toxin, or *S. aureus*, but not TNF alone, while OHI skin fibroblasts showed increased levels of cell death only after stimulation with alpha-toxin or *S. aureus* [Rows 61–65] [39,40].

Other cellular processes, such as proliferation, phagocytosis, or oxidative burst capacity, appeared largely unaffected in these patients [rows 66–71].

#### Functional studies

The subset of published variants was functionally evaluated *in vitro* predominantly in HEK293 cells, while V82I was modeled *in silico*. Assay data was available for 3 ORAS/OHI variants (G174Dfs*2, Y244C, EX6+2T>C), 4 ORAS-specific variants (M86I, W167S, L272P, G281R), 6 OHI-specific variants (E95*, D246V, P254S, R263Q, D268Tfs*6, N341D), and 1 DN-ORAS variant (C129S) (Figure 4, Table S4).

All tested missense variants resulted in normal *OTULIN* mRNA levels [Row 1], and nearly all in WT-like protein levels except for L272P and W199R [Row 2]. Protein levels for these latter two appeared to be reduced in some [13] but not in other assays [32,39], possibly as a consequence of reduced thermal stability. Stop-gain, frameshift and splice-altering variants all resulted in reduced or nearly absent protein levels.

*In silico* 3D structural modeling suggested that all variants examined led to aberrant protein conformations [Row 3], while 3/5 were also predicted to reduce protein thermal stability (W167S, L272P, G281R) [Row 4]. Predicted protein half-life was normal for 2 variants (M86I, W167S) [Row 5]. OTULIN interaction with LUBAC through HOIP was normal for 1 over-expressed frameshift variant (G174Dfs*2) and 2 missense (Y244C and L272P) variants [Row 6] [32]. By contrast, OTULIN binding affinity for M1-Ub chains was shown or predicted to be reduced for all but 2 DN-ORAS variants (C129A, C129S) [Row 7] [9].

A common consequence of almost all variants tested was the loss or reduction of OTULIN’s DUB activity with consequent accumulation of Met1-Ub chains and increased NF-**κ**B signaling, though there are some nuances in the data for Y244C (see Discussion). Two OHI variants (D246V and P254S) showed normal DUB activity and M1-Ub chain levels, but reduced inhibition of NF-**κ**B activity [Rows 8–9]. Presumed benign variant Q115H as well as the rare variant c.1033dup; p.(R345Kfs*4) showed WT-like levels of NF-**κ**B inhibition [Rows 10–12]. As opposed to the other frameshift variants (G174Dfs*2, D268Tfs*6, R345Kfs*4) appears to lead to WT level production of OTULIN protein. Therefore, this variant may be an isomorph, though its function was only assessed in a single assay and other assays might show other uninterrogated functional abnormalities.

Caspase-like, but not chymotryptic-like or tryptic-like, proteasome activity was found to be decreased in cells expressing C129A or L272 [Rows 13–15], while C129S expression in THP-1 cells increased some inflammatory gene expression (i.e. *IFNB1* and *IL6*, but not *IRF7* or *TNF*) [Rows 16–19]. This supports observations in patient cells suggesting activation of multiple streams of inflammatory signaling, including type I IFN.

## Discussion

### GenIA as a research and clinical tool

In this manuscript, we present a patient with a novel ORAS-related *OTULIN* variant and go on to systematically review OTULIN-related conditions using the GenIA database [20]. Generating a systematic review is a complex process that requires comprehensive and unbiased data mining in conjunction with harmonization and synthesis across multiple dimensions of relevant data. GenIA is a patient-centered, multidimensional IEI-specific database that enables aggregation and sophisticated data interrogation. Therefore, we populated GenIA with the information extracted from all papers published thus far reporting variants and patients with OTULIN-related conditions. GenIA confers rigor and efficiency to this process while maintaining case-specific nuances, thus serving as an ideal platform for unifying knowledge about genetically-driven immune disease. A potential limitation might be related to the fact that GenIA uses a fine-grained annotation scheme, which requires expert knowledge and manual effort, thus the annotation quality may vary depending on the curator’s expertise and consistency. The identification of redundancy across papers by the curator and the use of consensus nomenclature and ontological language available in GenIA enable the standardization of multiple connected layers of genetic, phenotypic and laboratory data (Figure 3A) to more accurately answer clinical and research questions.

It is important to note that an ongoing challenge of clinical data curation is distinguishing the true absence of a clinical feature from its not being interrogated, particularly without collateral communication from the authors. Moreover, some identical clinical features may be reported in different ways that can be difficult to reconcile statistically - for example, 3 patients described to have ‘skin rash’, ‘panniculitis’ or ‘neutrophilic dermatosis’ may actually share the same phenotype. This highlights an ongoing need for standardizing the way in which clinical data is collected and reported in research articles or disease-related databases.

Other databases, such as OMIM and ClinGen, could not be used for this review. OMIM generally only includes the first published articles and was last reviewed for OTULIN on 09/24/2022, and ClinGen still needs to publish curations for OTULIN.

### OTULIN-associated clinical and genetic features

OTULIN is currently associated with 3 clinically and pathophysiologically distinct disorders. Both biallelic LOF mutations and monoallelic dominant-negative mutations cause ORAS-like inflammatory phenotypes. Some symptoms, such as joint inflammation, are only reported in about 50% of cases, while others, such as hypergammaglobulinemia or GI involvement, may be under-reported. These have thus far only been identified in pediatric patients, with largely early-onset severe manifestations associated with significant diagnostic delays. On the other hand, *OTULIN* haploinsufficiency leads to incompletely penetrant immunodeficiency that manifests as susceptibility to invasive *S. aureus* infections, rather than simply an attenuated form of ORAS [39]. A number of individuals with these conditions are adults diagnosed retrospectively after their children are found to have AR ORAS. This recapitulates a paradigm seen with other genetic immune conditions such as X-linked chronic granulomatous disease, where differences in gene dosage may lead to different pathogenicity mechanisms and clinical outcomes [43]. From a management perspective, this also means that heterozygous parents or siblings of ORAS patients should not be assumed to be asymptomatic but should be carefully screened for infections. Finally, a recent report of TNF-responsive severe skin and soft tissue inflammation in a patient heterozygous for a predicted conservative substitution at a non-catalytic residue may constitute a novel DN-ORAS mutation, be an example of AR ORAS where the second mutation failed to be detected in *trans*, or constitute phenotypic expansion of the OHI phenotype. Discussion with the authors of the paper had suggested that the first possibility is currently most likely in the absence of functional data.

All the reported mutations thus far fall within the OTU domain that forms the bulk of the protein but no obvious genotype-phenotype correlations can be drawn from either the nature of the variant (i.e., missense, frameshift, nonsense, in-frame deletion) or its location. In other words, this information alone does not appear to help predict whether a particular variant is likely to be associated with any of the 3 known OTULIN-related conditions (ORAS, OHI, or DN-ORAS). However, limited evidence suggests that compound heterozygosity for hypomorphic mutations may lead to relatively later-onset disease. From the available experimental data available, it is clear that the impact of most mutations is more nuanced than total loss of the protein and all of its functions.

### Protein level effects

ORAS pathogenesis is thought to center on the inability of defective OTULIN to remove M1-Ub chains from I**κ**B and other key inflammatory substrates, resulting in increased activation of NF-**κ**B and other immune signaling pathways. However, both patient-based and *in vitro* studies suggest diverse forms of impact at the protein level, with diverse quantitative and qualitative downstream effects on Ub dynamics and specific pathways. While mRNA levels are rarely impacted, protein levels may be reduced or unstable, while changes in protein structure may lead to altered substrate interactions and/or reduced DUB activity. Moreover, OTULIN is also subject to phosphorylation, acetylation, and ubiquitination, so some mutations may also impact how OTULIN and its interactions are regulated by these PTMs.

OTULIN is also involved in a feedback mechanism whereby it binds to and promotes LUBAC activity by preventing the latter’s auto-ubiquitination [12]. Some divergent effects on LUBAC subunit levels have been seen in human cells, but it remains unclear if these observations are artefactual or reflections of true biology. Two ORAS patients (A023 and D049) showed reduced LUBAC subunit levels, which the authors proposed was a consequence of LUBAC down-regulation via proteasomal degradation in OTULIN-deficient patient fibroblasts to reduce the levels of M1-linked Ub and prevent activation of NF-**κ**B signaling (7). Stimulation by TNF-alpha appeared to increase LUBAC subunit levels in the B cells and fibroblasts of another ORAS patient (F069); this was attributed to enhanced LUBAC recruitment to the TNFR1 signaling complex (TNFR1-SC) (10).

### Ubiquitin dynamics

Some OHI variants (D246V and P254S) show ORAS-like effects in terms of reduced NF-**κ**B suppressive ability, but apparently normal DUB activity and M1-Ub chain levels. This suggests the potential for additional unexamined OTULIN targets and functions. OTULIN may exert tissue- and/or substrate-specific effects, so more subtle defects may require examination of tissue- and target-specific ubiquitination. OTULIN dysfunction may also be compensated for by the activities of other DUBs, such as CYLD. RIPK1 and TNFR1 are two OTULIN substrates whose activities are also regulated by K63 DUBs, such as A20 [44]. Though the impact of all OTULIN mutations on K63-Ub of relevant substrates has not been fully interrogated, one study noted that at least three ORAS/OHI mutations (L272P, Y244C, G174Dfs*2) had little to no impact on K63-linked RIPK1 or NEMO ubiquitination despite the increased abundance of linear M1-linked Ub in patient cells. PAMP and DAMP sensors such as NOD2, RIG-I and TLRs also funnel into downstream activation of NF-**κ**B, JAK-STAT, and other signaling pathways. Indeed, OTULIN has been shown to increase signaling downstream of NOD2 activation via the accumulation of M1-Ub on RIPK2 [45]. OTULIN has also been implicated in regulating other Ub-dependent processes such as Wnt signaling in angiogenesis and xenophagy [10,42], not to mention emerging Ub-independent functions at specific subcellular organelles - these roles may also contribute to disease pathogenesis [46].

In contrast to other OHI variants mentioned above, N341D leads to apparent abnormalities of M1-Ub binding and accumulation, but no downstream increases in NF-**κ**B signaling. Structural modeling suggested that this variant impacts catalytic triad coordination and altered interactions with WT M1-Ub - the catalytic Asn341 is replaced by a more negatively charged Asp, which would be expected to stabilize active conformation His339 to generate a more reactive enzyme. However, WT Met1-diUb serves as a poor substrate for this mutant protein, attributed to Coulombic charge repulsion in the catalytic center [9], likely leading to more complex and nuanced effects on target-specific deubiquitination. M1-Ub accumulation has also been suggested to capture some of the CYLD activity originally primed for K63-Ub removal, leading to the secondary accumulation of K63-Ub-decorated caveolin-1 complexes seen in some patient cells [39]. This accumulation is thought to play a role in α-toxin-induced cell death in OHI patients. Thus, pathogenesis in some OHI patients may be more related to generally disrupted Ub pool dynamics than the inability to suppress specific inflammatory signaling pathways.

Finally, OTULIN DUB activity is dependent on Cys129, a key conserved catalytic triad residue. Mutations at this site have been linked to a dominant-negative form of ORAS; specifically, *in vitro* studies show that co-expression of C129S and WT OTULIN in HEK293 cells leads to LUBAC-dependent linear Ub chain accumulation and consequent inability to suppress NF-κB activity [40]. Cells from DN-ORAS patients also phenotypically resemble those from ORAS patients in terms of increased M1-Ub chain accumulation on substrates, downstream expression of inflammatory cytokine genes (i.e. *TNF, IL6, IFNB1*), cell death, and type I IFN-activated gene signature. Both C129A and C129S mutant proteins have high affinity for M1-diUb but cannot cleave linear ubiquitin chains *in vitro*, so may act as catalytically-inactive, ‘decoy’ Ub-binding domains (UBDs) that compete with other M1-Ub-specific UBDs involved in regulating NF-κB signaling, in a manner resembling the effects of over-expressing the NEMO UBAN domain [47].

### Inflammatory signaling pathways

As linear Ub regulates diverse cellular processes, multiple inflammatory pathways contribute to ORAS. All of the ORAS-associated mutations tested *in vitro* except for Y244C (associated with ORAS and OHI) led to some evidence of increased NF-**κ**B activity at baseline (Figure 5). *In vitro* over-expression of Y244C in HEK293 cells by several groups showed WT-like to mildly increased levels of NF-**κ**B activity, target-specific linear deubiquitination, and M1-Ub accumulation at baseline, though TNF stimulation uncovered severely defective NF-**κ**B suppression [32,39,40], also seen in patient leukocytes and fibroblasts [32]. This suggests that stimulation using pathway-appropriate cytokines may sometimes be required to uncover defects not seen at baseline. In other words, some ORAS-related mutations may lead to baseline constitutive activation, while others may only show stimuli-induced hyperactivation. As more patients are identified, it will be interesting to see if these differences correspond to differences in clinical presentation.

In addition, IL-1β stimulation of PBMCs from ORAS patients can also lead to the accumulation of linear ubiquitinated NEMO, TNFR1, RIPK1, ASC and high-molecular weight M1-Ub aggregates and pro-inflammatory cytokine production. Indeed, M1-Ub chain formation on ASC contributes to NLRP3 inflammasome formation and downstream caspase-1 activation [48,49]. Thus, OTULIN may regulate this and other LUBAC-dependent contributions to inflammasome activation (38).

Finally, some ORAS patient cells also show strong signatures of JAK-STAT and IFN activation. In OTULIN-deficient patients, M1-Ub chain accumulation has been found to cause defects in immunoproteasome assembly and function in a manner reminiscent of the PRAAS/CANDLE mutations with similarly upregulated type I IFN signaling [19]. However, linear STAT1 ubiquitination has also been found to block interaction with IFNα/β receptor 2 (IFNAR2) [50], so OTULIN may make both positive and negative contributions to type I IFN activation.

### Cell-type specific effects

As for other innate immune genes, interpretation of mutational impact has been confounded by potentially divergent cell-type specific effects, also reflected in differences seen with conditional vs global knockout mouse models. OTULIN interacts with and performs linear deubiquitination of proteasome subunits. Differential effects on proteasome function have been shown for hematopoietic and non-hematopoietic cells from ORAS patients. Both patient PBMCs and fibroblasts show reduced caspase-like proteasome activity, but only reduced tryptic-like and chymotryptic-like proteasome activity was reported for PBMCs, suggesting a more immunoproteasome-specific effect.

Keratinocyte-specific *Otulin* KO mice appear to show *enhanced* TNF-driven cell death, leading to inflammatory skin lesions via increased IL-1β and type I IFN signaling (37). This is similar to reports of increased TNF-dependent NF-κB activation seen in the hematopoietic cells but not skin fibroblasts of ORAS patients. In contrast, the latter appear to show increased cell death as a consequence of impaired rather than hyperactive responses to TNF signaling [35]. In the conditional KO mice, TNF signaling is thought to promote cell death via formation of 1) an apoptosis-inducing complex involving RIPK1-FADD (Fas-associated death domain) and caspase-8 (Complex II) or 2) a RIPK1-dependent necroptosis-inducing complex (necrosome) that acts via RIPK3-mediated MLKL (mixed lineage kinase domain-like) phosphorylation. Indeed, the combined loss of cell death mediators Caspase-8 (for apoptosis) and RIPK3 (for necroptosis) appears to ameliorate the TNFR1- and RIPK1-dependent lethality seen in mouse embryos with catalytically inactive OTULIN [12]. However, even these partially rescued mice die perinatally, ostensibly from enhanced RIPK1-dependent type I IFN production. As for other forms of monogenic immune disease, this data suggests that too much or too little signaling in one pathway may have similar clinical and cellular consequences. It also further highlights the complex crosstalk that exists between the multiple pathways contributing to ORAS pathogenesis.

For heterozygous patients, OTULIN haploinsufficiency appears to impair cell-intrinsic immunity to the major *S. aureus* virulence factor, α-toxin, conferring susceptibility to α-toxin-induced fibroblast death. While this may involve some of the M1- vs K63-Ub pool disruptions mentioned above, another possibility is its direct regulation of LUBAC-dependent linear ubiquitination on bacteria, which can activate xenophagy and local NF-κB signaling [51,52]. Both pathogen-induced cell death and overly robust host inflammatory responses may contribute to the morbidity seen in OHI patients, so it is still too early to rule out the possibility of inflammatory phenotypes associated with this condition. In addition to pathogen exposure, levels of naturally elicited α-toxin-neutralizing antibodies are shown to contribute to the observed variable expressivity and reduced penetrance since levels of these antibodies may decline with age [39].

### Implications for management

Current management for ORAS is symptom-focused, with the goal of reducing inflammation and preventing organ and tissue damage. As for other rare genetically-driven immune diseases, no consensus guidelines currently exist for ORAS, so immunomodulation choice for ORAS is often dictated by disease severity, local resource availability, and provider preferences. However, given the importance of upstream TNF signaling in OTULIN-related pathogenesis, it is not surprising that most patients show positive clinical responses to TNF inhibition (Figure 3D). Due to the suspected contributions from inflammasome and JAK-STAT signaling, IL-1 inhibition and JAK inhibition have also been tried with positive effects in some patients. Given the significant pathophysiological contribution from myeloid cells, the use of lymphocyte-targeting immunomodulation may be less effective.

The ability of some patients to partially respond to colchicine and anakinra highlights the importance of dissecting OTULIN’s pleiotropic, cell-specific functions, which may result in clinically relevant tissue-specific outcomes difficult to assess from peripheral blood samples alone. It also highlights the importance of deep phenotyping when describing clinical responses to therapies, as only a subset of clinical phenotypes may respond to immunomodulation, particularly in various subsets of hematopoietic and non-hematopoietic cells. For example, IL-1β neutralization appears to be most helpful for treating ORAS-related cutaneous inflammation, likely via inhibition of the increased cell death and caspase-dependent IL-1 signaling seen in skin fibroblasts.

For disorders arising from defects in ubiquitous cellular signaling processes, there is always the concern that HSCT may not repair disease manifestations in non-hematopoietic cells, though it may help curb feed-forward inflammatory signaling. Indeed, the inflammatory phenotypes of OTULIN LOF in adult mice are not entirely rescued by reconstitution with WT bone marrow, suggesting the relevance of OTULIN activity in non-hematopoietic cells [12]. In terms of related disorders, patients with NEMO mutations have been reported with ongoing post-HSCT colitis [53], while the gastrointestinal inflammation in one RIPK1-deficient patient appears to have been resolved by HSCT [54]. However, there have been cases of successful HSCT reported for SAID patients [55,56]. The experience of patient D049 and the possibility that some OTULIN-related inflammatory mechanisms may be hematopoietic lineage-specific (i.e., immunoproteasome dysregulation) suggests that HSCT should continue to be considered for ORAS patients. Timely molecular diagnosis may also lead to timely HSCT with fewer comorbidities and better outcomes.

From an infectious perspective, few ORAS or DN-ORAS patients are reported to have a clinical history of infection or known culture positivity. As for our patient, most infectious workups were performed and antimicrobials given in the setting of unexplained systemic inflammation, but antimicrobial prophylaxis is rarely considered otherwise and detailed evaluations for immunodeficiency have not been reported. In particular, potential susceptibility to Mycobacteria could be a concern with dysregulated NF-**κ**B and IFN signaling, but non-hematopoietic cells may also harbor tissue-tropic pathogen susceptibilities worth investigating.

## Figures and Tables

**Figure 1.** Case presentation. (**A**) Pedigree for our patient case with parental segregation of the novel *OTULIN* mutation shown. The black-filled symbol represents the affected patient, while the white symbol represents unaffected parents. (**B**) Images from the deceased patient harboring the novel homozygous OTULIN mutation. (**C**) Sanger sequencing electropherograms showing the nucleotide sequence change and below, the predicted codon and amino acid sequence change in the OTULIN protein. (**D**) *In silico* 3D modeling of the missense change: interactions established between Trp199 or Arg199 and the residues of OTULIN. Hydrophobic interactions are depicted in red, aromatic in blue, pi-pi in orange, carbon-pi in magenta, metsulfur-pi in yellow, amide-ring in hot pink, and hydrogen bonds in salmon. (**E**) Western blotting of protein extracts from HEK293T cells transfected with an empty plasmid, LUBAC plasmids (equal amounts of HOIP, HOIL-1, SHARPIN), OTULIN wild type (WT) or with a mutant plasmid (W199R or L272P) using antibodies against OTULIN, SHARPIN, HOIL-1, HOIP. (**F**) WB of co-immunoprecipitation assay using protein extracts from HEK293 cells shown in (E) that additionally express HA-tagged Ubiquitin and NEMO. NEMO was used as bait to pull down the complex. Immunoblot shows the presence and relative abundance of NEMO, OTULIN and Ub chains. (**G**) Quantification of protein expression relative to GAPDH and to OTULIN-WT levels from WB images in (E) and Figure S2D using ImageJ software. See Figure S2F for quantification of ubiquitination (Ub-HA) (**H**) Dual-luciferase assay on the HEK293T cells used in (E) additionally transfected with equal amounts of NF-kB driven luciferase reporter plasmid/renilla control plasmid, after 18 hr in culture. The fold change of Firefly luciferase versus Renilla luciferase was normalized to cells transfected with an empty vector. Results of three independent experiments are shown. Error bars depict standard deviations from triplicate samples.

**Figure 2.** Systematic literature review of OTULIN disease-causing variants. (**A**) Total number of individuals (patients and family members) according to the number of times they were reported or mentioned in an article. Below, total number of disease-associated OTULIN variants found in GenIA vs OMIM and ClinVar. (**B**) Upset plot shows how many patients had genetic, clinical, functional, and lab data available across all articles. It also shows how many patients had a combination of clinical and genetic data; clinical, genetic and lab data; or all 4 datasets available. (**C**) Schematic representation of all OTULIN disease-causing variants displayed along OTULIN’s gene/cDNA and protein sequences. Variants associated with ORAS/DN-ORAS are shown above the respective cDNA and protein sequences and below variants associated with OHI. Each dot represents a patient.

**Figure 3.** Clinical and management data. (**A**) Schematic representation of the cardinal symptoms found to be present or absent in OTULIN-related diseases (ORAS, DN-ORAS and OHI). Human figure template borrowed and modified for the purposes of this paper. (**B**) Graph showing the ages at which the different ORAS patients began to present clinically (AFM, age at first manifestation), the ages at which they were genetically studied or diagnosed (ADx), the ages at which they died (ADeath), and the age at which one patient received HSCT. Circles indicate females, and triangles indicate males. (**C**) Graph showing disease penetrance in OHI for confirmed heterozygotes or confirmed plus presumed/obligate heterozygotes for pathogenic variants in *OTULIN*. (**D**) Matrix showing the different therapies ORAS patients received and their respective responses/outcomes. *Unspecified* means that outcome was not explicitly mentioned by authors.

**Figure 4.** *In vitro* (or *in silico*) functional consequences of OTULIN mutants. Matrix showing the assays used and respective outcomes for all reported *OTULIN* variants, both naturally occurring and artificially generated. In bold are those variants with some causal association to human OTULIN-related diseases. A box containing multiple colors indicates that multiple experimental data points were generated for an assay, either in different studies or in the same study using different conditions. For the full details associated with each assay, please see Table S4.

**Figure 5.** OTULIN function and dysfunction. Simplified and schematic representation of OTULIN’s role as a DUB within the NF-**κ**B signaling pathway in WT and ORAS cells. Defective OTULIN cannot remove Met1-Ub chains from I**κ**Bs, and this leads to increased phosphorylation of I**κ**Kalpha/beta, I**κ**B-alpha, and p65-NF-**κ**B following TNF signaling, with consequent activation of NF-**κ**B signaling. Presumed pathway alterations in mutant cells are shown in bold.

**Table 1.** All individuals with OTULIN pathogenic variants (n=56)

SubjectID	Tree_pos	GenIA_UID	GenIA_FamID	Sex	Dis_status	Diagnosis	AAD	Origin	Population	Pub_codes	Variant(s)	Zygosity(s)	Relevance	Variant_IDs
**A006**	II.2	104561	215551	F	Unaffected	NA	NA	Pakistan	Pakistani	PMID:27523608 [Fam.1:I.2]; PMID:27559085 [Fam.1:II.5]	L272P	OC	No	1415
**A018**	IV.1	104567	215551	M	Unaffected	NA	NA	Pakistan	Pakistani	PMID:27523608 [Fam.1:IV.1(IV:1)]; PMID:27559085 [Fam.1:III.5]; PMID:35587511 [Fam.G:I.2(2)]	L272P	HET	No	1415
**A019**	IV.2	104573	215551	F	Unaffected	NA	NA	Pakistan	Pakistani	PMID:27523608 [Fam.1:IV.2(IV:2)]; PMID:27559085 [Fam.1:IV.6]; PMID:35587511 [Fam.G:I.1(1)]	L272P	HET	No	1415
**A020**	IV.3	104564	215551	M	Unaffected	NA	NA	Pakistan	Pakistani	PMID:27523608 [Fam.1:III.3]; PMID:27559085 [Fam.1:III.2]	L272P	OC	No	1415
**A021**	IV.4	104563	215551	F	Unaffected	NA	NA	Pakistan	Pakistani	PMID:27523608 [Fam.1:III.4]; PMID:27559085 [Fam.1:III.1]	L272P	OC	No	1415
**A023**	V.2	104554	215551	M	Affected	ORAS (AR)	NA	Pakistan	Pakistani	PMID:27523608 [Fam.1:V.2(V:2)]; PMID:27559085 [Fam.1:V.2(P1)]; PMID:35587511 [Fam.G:II.2(4)]; PMID:34797715 [P1]	L272P	HOM	Yes, alone	1415
**A024**	V.3	104574	215551	F	Unaffected	NA	NA	Pakistan	Pakistani	PMID:27523608 [Fam.1:V.3]; PMID:27559085 [Fam.1:V.1]; PMID:35587511 [Fam.G:II.1(3)]	L272P	HET	No	1415
**A027**	V.6	104568	215551	F	Affected	ORAS (AR)	1.3	Pakistan	Pakistani	PMID:27523608 [Fam.1:IV.3(IV:3)]; PMID:32231246 [IV:3(IV.3)]; PMID:27559085 [Fam.1:IV.1(P4)]	L272P	HOM	Yes, alone	1415
**A028**	V.7	104570	215551	F	Affected	ORAS (AR)	5.0	Pakistan	Pakistani	PMID:27523608 [Fam.1:IV.4(IV:4)]; PMID:27559085 [Fam.1:IV.3(NA)]	L272P	HOM	Yes, alone	1415
**B033**	I.1	104577	215552	M	Unaffected	NA	NA	Turkey	Turkish	PMID:27559085 [Fam.2:I.2]; PMID:35587511 [Fam.I:I.2(2)]	Y244C	HET	No	1416
**B034**	I.2	104576	215552	F	Affected	OHI (AD)	NA	Turkey	Turkish	PMID:27559085 [Fam.2:I.1]; PMID:35587511 [Fam.I:I.1(1)]	Y244C	HET	Yes, alone	1416
**B035**	II.1	104575	215552	F	Affected	ORAS (AR)	NA	Turkey	Turkish	PMID:27559085 [Fam.2:II.1(P2)]; PMID:35587511 [Fam.I:II.2(4)]; PMID:34797715 [P2]	Y244C	HOM	Yes, alone	1416
**B036**	II.2	104578	215552	F	Unaffected	NA	NA	Turkey	Turkish	PMID:27559085 [Fam.2:II.2]; PMID:35587511 [Fam.I:II.1(3)]	Y244C	HET	No	1416
**C037**	I.1	104582	215553	M	Affected	OHI (AD)	NA	Turkey	Turkish	PMID:27559085 [Fam.3:I.2]; PMID:35587511 [Fam.H:I.2(2)]	G174Dfs*2	HET	Yes, alone	1417
**C038**	I.2	104581	215553	F	Unaffected	NA	NA	Turkey	Turkish	PMID:27559085 [Fam.3:I.1]; PMID:35587511 [Fam.H:I.1(1)]	G174Dfs*2	HET	No	1417
**C039**	II.1	104580	215553	F	Affected	ORAS (AR)	NA	Turkey	Turkish	PMID:27559085 [Fam.3:II.1(P3)]; PMID:35587511 [Fam.H:II.1(3)]; PMID:34797715 [P3]	G174Dfs*2	HOM	Yes, alone	1417
**D042**	II.1	104585	215554	M	Unaffected	NA	NA	Israel	Arab	PMID:30804083 [Fam.Patient:I.2]	G281R	OC	No	1419
**D044**	II.3	104586	215554	M	Unaffected	NA	NA	Israel	Arab	PMID:30804083 [Fam.Patient:I.3]	G281R	OC	No	1419
**D046**	III.1	104588	215554	M	Unaffected	NA	NA	Israel	Arab	PMID:30804083 [Fam.Patient:II.1]; PMID:35587511 [Fam.K:I.2(2)]	G281R	HET	No	1419
**D047**	III.2	104589	215554	F	Unaffected	NA	NA	Israel	Arab	PMID:30804083 [Fam.Patient:II.2]; PMID:35587511 [Fam.K:I.1(1)]	G281R	HET	No	1419
**D048**	IV.1	104590	215554	F	Unaffected	NA	NA	Israel	Arab	PMID:30804083 [Fam.Patient:III.1]; PMID:35587511 [Fam.K:II.1(3)]	G281R	HET	No	1419
**D049**	IV.2	104583	215554	F	Affected	ORAS (AR)	NA	Israel	Arab	PMID:30804083 [Patient(III.2)]; PMID:35587511 [Fam.K:II.2(4)]	G281R	HOM	Yes, alone	1419
**E057**	III.1	104600	215555	M	Unaffected	NA	NA	Iran	Iranian	PMID:30796585 [Fam.Patient:III.2]	EX6+2T>C	OC	No	1418
**E059**	III.3	104601	215555	M	Unaffected	NA	NA	Iran	Iranian	PMID:30796585 [Fam.Patient:III.3]	EX6+2T>C	OC	No	1418
**E061**	IV.1	104603	215555	F	Unaffected	NA	NA	Iran	Iranian	PMID:30796585 [Fam.Patient:IV.1]; PMID:35587511 [Fam.J:I.1(1)]	EX6+2T>C	HET	No	1418
**E062**	IV.2	104604	215555	M	Affected	OHI (AD)	NA	Iran	Iranian	PMID:30796585 [Fam.Patient:IV.2]; PMID:35587511 [Fam.J:I.2(2)]	EX6+2T>C	HET	Yes, alone	1418
**E064**	V.2	104606	215555	F	Unaffected	NA	NA	Iran	Iranian	PMID:30796585 [Fam.Patient:V.2]; PMID:35587511 [Fam.J:II.1(3)]	EX6+2T>C	HET	No	1418
**E065**	V.3	104592	215555	F	Affected	ORAS (AR)	0.7	Iran	Iranian	PMID:30796585 [Patient(V.3)]; PMID:35587511 [Fam.J:II.3(5)]	EX6+2T>C	HOM	Yes, alone	1418
**F066**	I.1	104613	215557	M	Unaffected	NA	NA	Germany	Greek	PMID:35170849 [Fam.Patient:I.1]	W167S & M86I	WT & HET	No & No	1421,1420
**F067**	I.2	104614	215557	F	Unaffected	NA	NA	Germany	Greek	PMID:35170849 [Fam.Patient:I.2]	W167S & M86I	HET & WT	No & No	1421,1420
**F069**	II.2	104612	215557	M	Affected	ORAS (AR)	NA	Germany	Greek	PMID:35170849 [Patient(II.2)]	W167S & M86I	HET & HET	Yes, combined	1421,1420
**G072**	I.2	104620	215558	F	Affected	OHI (AD)	NA	Netherlands	Dutch	PMID:35587511 [Fam.A:I.1(1)]	D246V	HET	Yes, alone	1424
**G073**	II.1	104623	215558	M	Unaffected	NA	NA	Netherlands	Dutch	PMID:35587511 [Fam.A:II.1(3)]	D246V	HET	No	1424
**G074**	II.2	104624	215558	F	Unaffected	NA	NA	Netherlands	Dutch	PMID:35587511 [Fam.A:II.2(4)]	D246V	HET	No	1424
**G075**	II.3	104619	215558	M	Affected	OHI (AD)	19.0	Netherlands	Dutch	PMID:35587511 [Fam.A:II.3(5)]	D246V	HET	Yes, alone	1424
**H077**	I.2	104645	215563	F	Affected	OHI (AD)	Unk.	Argentina	Argentinian	PMID:35587511 [Fam.B:I.1(1)]	E95*	OC	Likely	1425
**H079**	II.2	104647	215563	F	Affected	OHI (AD)	Unk.	Argentina	Argentinian	PMID:35587511 [Fam.B:II.1(3)]	E95*	OC	Likely	1425
**H080**	III.1	104644	215563	M	Affected	OHI (AD)	NA	Argentina	Argentinian	PMID:35587511 [Fam.B:III.2(5)]	E95*	HET	Yes, alone	1425
**H082**	III.3	104650	215563	M	Affected	OHI (AD)	NA	Argentina	Argentinian	PMID:35587511 [Fam.B:III.3(6)]	E95*	HET	Yes, alone	1425
**H083**	IV.1	104651	215563	M	Affected	OHI (AD)	NA	Argentina	Argentinian	PMID:35587511 [Fam.B:IV.1(7)]	E95*	HET	Yes, alone	1425
**I088**	II.2	105133	215564	F	Unaffected	NA	NA	Mexico	Mexican	PMID:35587511 [Fam.C:II.1(3)]	D268Tfs*6	HET	No	1782
**I089**	III.1	104653	215564	M	Affected	OHI (AD)	19.0	Mexico	Mexican	PMID:35587511 [Fam.C:III.1(5)]	D268Tfs*6	HET	Yes, alone	1782
**J091**	I.2	104657	215565	F	Unaffected	NA	NA	France	French	PMID:35587511 [Fam.D:I.1(1)]	N341D	HET	No	1426
**J094**	II.3	104656	215565	M	Affected	OHI (AD)	NA	France	French	PMID:35587511 [Fam.D:II.3(5)]	N341D	HET	Yes, alone	1426
**K095**	I.1	104663	215566	M	Unaffected	NA	NA	Belgium	Belgian	PMID:35587511 [Fam.E:I.2(2)]	P254S	HET	No	1427
**K097**	II.1	104664	215566	F	Unaffected	NA	NA	Belgium	Belgian	PMID:35587511 [Fam.E:II.1(3)]	P254S	HET	No	1427
**K098**	II.2	104665	215566	F	Affected	OHI (AD)	NA	Belgium	Belgian	PMID:35587511 [Fam.E:II.2(4)]	P254S	HET	Yes, alone	1427
**K099**	II.3	104661	215566	F	Affected	OHI (AD)	NA	Belgium	Belgian	PMID:35587511 [Fam.E:II.3(5)]	P254S	HET	Yes, alone	1427
**L102**	II.1	104666	215567	M	Affected	OHI (AD)	NA	France	French	PMID:35587511 [Fam.F:II.1(3)]	R263Q	HET	Yes, alone	1428
**M105**	I.1	105136	215649	M	Unaffected	NA	NA	Morocco	Moroccan	GRID:903 [Fam.M:I.1(105)]	W199R	HET	No	1791
**M106**	I.2	105137	215649	F	Unaffected	NA	NA	Morocco	Moroccan	GRID:903 [Fam.M:I.2(106)]	W199R	HET	No	1791
**M107**	II.1	105135	215649	F	Affected	ORAS (AR)	0.2	Morocco	Moroccan	GRID:903 [Fam.M:II.1(107)]	W199R	HOM	Yes, alone	1791
**N110**	II.1	105162	215658	M	Affected	DN-ORAS (AD)	NA	Australia	Australian	GRID:912 [Fam.1:Patient 1(II.1)]	C129S	HET	Yes, alone	1806
**O113**	II.1	105276	215684	F	Affected	DN-ORAS (AD)	NA	Saudi Arabia	Saudi	GRID:912 [Fam.2:II.1(Patient 2)]	C129S	HET	Yes, alone	1806
**P115**	I.2	105415	215763	F	Unaffected	NA	NA	Netherlands	NA	PMID:38129331 [Fam.case:I.2]	A240V	HET	Unlikely	2082
P116	II.1	105413	215763	F	Affected	OHI (AD)	NA	Netherlands	NA	PMID:38129331 [case(II.1)]	A240V	HET	Yes, alone	2082

GenIA_UID: Unique subject identifier in GenIA’s database; GenIA_FamID: Unique family identifier in GenIA’s database; AAD: age at death in years; ORAS: OTULIN-related autoinflammatory syndrome; OHI: OTULIN Haploinsufficiency (susceptibility to severe S. aureus infections); DN-ORAS: Dominant Negative OTULIN-related autoinflammatory syndrome); AR: autosomal recessive; AD: autosomal dominant; OC: Obligate or presumed heterozygous carrier

**Table 2.** All reported (and novel) OTULIN variants

Var. ID	var. name	Chrom. change	Exon Intron	CDS change	Prot. change	Exon offset	Var. type	Var. class	Var. effect	dbSNP	ClinVar class.	OMIM id	Pat. count	gnomAD_alleles_exomes	gnomAD_alleles_genomes	MaxEntScan	dbscSNV	Refs
**1784**	Q8*	5–14664847-C-T	EX1	c.22C>T	p.Gln8Ter	NA	stop gained	LP	**Not tested**	NA	pathogenic	NA	0	NA	NA	NA	NA	Clinvar
**1788**	Q40R	5–14664944A-G	EX1	c.119A>G	p.Gln40Arg	NA	missense	LB	**Not tested**	rs750815369	uncertain_significance(1), Likely benign(1)	NA	0	0/100	131/150292	NA	NA	[38]
**1422**	V82I	5–14678695G-A	EX3	c.244G>A	p.Val82Ile	NA	missense	VUS	Not tested*	rs555528904	uncertain_si gnificance	NA	0	37/223872	10/151504	NA	NA	[38]
**1420**	**M86I**	5–14678709G-A	EX3	c.258G>A	p.Met86Ile	NA	missense	P	LOF - Hypomorphic	NA	pathogenic	615712 #0009	1	NA	NA	NA	NA	[37]
**1813**	Y91F	5–14678723A-T	EX3	c.272A>T	p.Tyr91Phe	NA	missense	LP	LOF	NA	NA	NA	0	NA	NA	NA	NA	[9]
**1425**	E95*	5–14678734G-T	EX3	c.283G>T	p.Glu95Ter	NA	stop gained	P	LOF - Amorphic	NA	risk factor	615712 #0005	5	NA	NA	NA	NA	[39]
**1812**	W96A	5–14678737-TG-GC	EX3	c.286_287delinsGC	p.Trp96Ala	NA	missense	LP	LOF - Amorphic	NA	NA	NA	0	NA	NA	NA	NA	[9]
**1783**	EX4–2A>G	5–14681462A-G	IN3	c.325–2A>G	NA	2	splice acceptor	LP	**Not tested**	rs1553995945	likely_patho genic	NA	0	NA	NA	7.955	0.9999 0.916	ClinVar
**1423**	Q115H	5–14681484G-T	EX4	c.345G>T	p.Gln115His	NA	missense	B	**Isomorphic**	rs147790160	benign	NA	0	1633/247450	814/152174	NA	NA	[38,39]
**1780**	C129A	5–14681524-TG-GC	EX4	c.385_386delinsGC	p.Cys129Ala	NA	missense	LP	LOF - Amorphic	NA	NA	NA	0	NA	NA	NA	NA	[9,19,35,37,40]
**1806**	**C129S**	5–14681525G-C	EX4	c.386G>C	p.Cys129Ser	NA	missense	P	**DN-LOF - Antimorphic**	NA	NA	NA	1	NA	NA	NA	NA	[40]
**1883**		5–14681524T-A		c.385T>A						NA	NA	NA	1	NA	NA	NA	NA	[40]
**1785**	P147S	5–14681578-C-T	EX4	c.439C>T	p.Pro147Ser	NA	missense	LB	**Not tested**	rs371959714	benign	NA	0	367/247658	84/152238	NA	NA	[38]
**1786**	M155L	5–14681602A-T	EX4	c.463A>T	p.Met155Leu	NA	missense	LB	**Not tested**	rs11953822	benign	NA	0	1137/237202	2873/152234	NA	NA	[38]
**1421**	**W167S**	5–14687552G-C	EX5	c.500G>C	p.Trp167Ser	NA	missense	P	LOF - Hypomorphic	NA	pathogenic	615712 #0010	1	NA	NA	NA	NA	[37]
**1417**	G174Dfs*2	5–14687568AC-A	EX5	c.517del	p.Gly174Aspf sTer2	NA	frameshift	P	LOF - Amorphic	rs886037886	pathogenic, risk_factor	615712 #0002	2	NA	NA	NA	NA	[32,38,39]
**1787**	V185F	5–14687605G-T	EX5	c.553G>T	p.Val185Phe	NA	missense	VUS	**Not tested**	rs867617260	uncertain_si gnificance	NA	0	NA	NA	NA	NA	[38]
**1791**	**W199R**	5–14690039T-A	EX6	c.595T>A	p.Trp199Arg	1	missense	LP	**LOF - Amorphic**	NA	NA	NA	1	NA	NA	NA	0.7205 0.67	CCI - this study
**1790**	L202F	5–14690050G-C	EX6	c.606G>C	p.Leu202Phe	NA	missense	VUS	Not tested	rs747025364	NA	NA	0	1/249070	NA	NA	NA	[38]
**2082**	**A240V**	5–14690163-C-T	EX6	c.719C>T	p.Ala240Val	NA	missense	LP	**Not tested**	NA	NA	NA	1	NA	NA	NA	NA	[41]
**1416**	**Y244C**	5–14690175A-G	EX6	c.731A>G	p.Tyr244Cys	NA	missense	P	LOF - Hypomorphic	rs886037887	risk_factor, pathogenic	615712 #0003	2	NA	NA	NA	NA	[19,32,38,39]
**1424**	**D246V**	5–14690181A-T	EX6	c.737A>T	p.Asp246Val	NA	missense	P	LOF - Hypomorphic	NA	Risk factor	615712 #0004	2	NA	NA	NA	NA	[39]
**1427**	**P254S**	5–14690204-C-T	EX6	c.760C>T	p.Pro254Ser	NA	missense	P	LOF - Hypomorphic	NA	NA	NA	2	NA	NA	NA	NA	[39]
**1808**	L259E	5–14690219-CTT-GAG	EX6	c.775_777delinsGAG	p.Leu259Glu	NA	missense	LP	LOF - Amorphic	NA	NA	NA	0	NA	NA	NA	NA	[9]
**1809**		5–14690219-CTT-GAA		c.775_777delinsGAA														
**1428**	**R263Q**	5–14690232G-A	EX6	c.788G>A	p.Arg263Gln	NA	missense	P	LOF - Amorphic	rs1332823115	NA	NA	1	1/249258	NA	NA	NA	[39]
**1782**	D268Tfs*6	5–14690245TG-T	EX6	c.802del	p.Asp268Thrf sTer6	NA	frameshift	P	LOF - Amorphic	NA	risk factor	615712 #0006	1	NA	NA	NA	NA	[39]
**1415**	**L272P**	5–14690259-T-C	EX6	c.815T>C	p.Leu272Pro	NA	missense	P	LOF - Amorphic	rs886037885	pathogenic	615712 #0001	3	NA	NA	NA	NA	[13,19,32,33,36–39]
**1419**	**G281R**	5–14690285G-A	EX6	c.841G>A	p.Gly281Arg	NA	missense	P	LOF - Hypomorphic	NA	pathogenic	615712 #0008	1	NA	NA	NA	NA	[35,38,39]
**1418**	**EX6+2T>C**	5–14690310-T-C	IN6	c.864+2T>C	(p.Trp199-Gln288del)	2	splice donor	P	LOF - Amorphic	NA	pathogenic	615712 #0007	2	NA	NA	7.754	0.9961 0.712	[34,39]
**1789**	N311S	5–14692921A-G	EX7	c.932A>G	p.Asn311Ser	NA	missense	LB	**Not tested**	rs9312870	benign	NA	0	1255/249576	2843/152114	NA	NA	[38]
**1810**	E314R	5–14692929-GA-CG	EX7	c.940_941delinsCG	p.Glu314Arg	NA	missense	LP	LOF	NA	NA	NA	0	NA	NA	NA	NA	[9]
**1811**		5–14692929-GA-AG		c.940_941delinsAG						NA	NA	NA	0	NA	NA	NA	NA	
**1807**	D336A	5–14692996A-C	EX7	c.1007A>C	p.Asp336Ala	NA	missense	LP	LOF	NA	NA	NA	0	NA	NA	NA	NA	[9]
**1814**	H339A	5–14693004-CA-GC	EX7	c.1015_1016delinsGC	p.His339Ala	NA	missense	LP	LOF - Amorphic	NA	NA	NA	0	NA	NA	NA	NA	[9]
**1426**	**N341D**	5–14693010A-G	EX7	c.1021A>G	p.Asn341Asp	NA	missense	P	LOF - Hypomorphic	NA	NA	NA	1	NA	NA	NA	NA	[9,39]
**1815**	N341A	5–14693010-AA-GC	EX7	c.1021_1022delinsGC	p.Asn341Ala	NA	missense	LP	LOF - Amorphic	NA	NA	NA	0	NA	NA	NA	NA	[9]
**1799**	R345Kfs*4	5–14693021C-CA	EX7	c.1033dup	p.Arg345LysfsTer4	NA	frameshift	LB	**Possibly Isomorphic**	rs746946210	NA	NA	0	25/248100	3/152190	NA	NA	[39]

Variant’s c. and p. positions are given according to OTULIN’s canonical transcript ENST00000284274.5. Chrom. Change refers to GRCh38/hg38 assembly. In bold OTULIN-related disease s variants.

Var. class (variant classification): P: pathogenic, LP: likely pathogenic, VUS: Variant of Uncertain Significance, LB: Likely Benign, B: Benign.

Var. effect (variant effect or functional consequence): LOF - loss of function, DN-LOF - dominant negative loss of function. NA - not available. OC - obligate/presumed carrier

Source:ClinVar submission; ORAS patient (this study)

## Data Availability

The data supporting this study’s findings are available from the corresponding author upon request.
